# Phylogenetic Placement of Isolates Within the Trans-Eurasian Clade A.Br.008/009 of *Bacillus anthracis*

**DOI:** 10.3390/microorganisms7120689

**Published:** 2019-12-12

**Authors:** Markus Antwerpen, Wolfgang Beyer, Olga Bassy, María Victoria Ortega-García, Juan Carlos Cabria-Ramos, Gregor Grass, Roman Wölfel

**Affiliations:** 1 Bundeswehr Institute of Microbiology (IMB), 80937 Munich, Germany; markusantwerpen@bundeswehr.org (M.A.); romanwoelfel@bundeswehr.org (R.W.); 2 Department Livestock Infectiology and Environmental Hygiene, University of Hohenheim, 70599 Stuttgart, Germany; wolfgang.beyer@uni-hohenheim.de; 3 Chemical, Biological, Radiological and Nuclear (CBRN) Defence Department, Campus La Marañosa, Instituto Nacional de Técnica Aeroespacial INTA, 28330 San Martín de la Vega (Madrid), Spain; obassy@isdefe.es (O.B.); ortegagmv@inta.es (M.V.O.-G.); cabriarjc@inta.es (J.C.C.-R.)

**Keywords:** Anthrax, *Bacillus anthracis*, genotyping, genome-sequencing, phylogeny

## Abstract

The largest phylogenetic lineage known to date of the anthrax pathogen *Bacillus anthracis* is the wide-spread, so-called Trans-Eurasian clade systematically categorized as the A.Br.008/009 group sharing two defining canonical single-nucleotide polymorphisms (canSNP). In this study, we genome-sequenced a collection of 35 *B. anthracis* strains of this clade, derived from human infections, animal outbreaks or soil, mostly from European countries isolated between 1936 and 2008. The new data were subjected to comparative chromosomal analysis, together with 75 *B. anthracis* genomes available in public databases, and the relative placements of these isolates were determined within the global phylogeny of the A.Br.008/009 canSNP group. From this analysis, we have detected 3754 chromosomal SNPs, allowing the assignation of the new chromosomal sequences to established sub-clades, to define new sub-clades, such as two new Spanish, one Bulgarian or one German group(s), or to introduce orphan lineages. SNP-based results were compared with that of a multilocus variable number of tandem repeat analysis (MLVA). This analysis indicated that MLVA typing might provide additional information in cases when genomics yields identical genotypes or shows only minor differences. Introducing the delayed mismatch amplification assay (DMAA) PCR-analysis, we developed a cost-effective method to interrogate for a set of ten phylogenetically informative SNPs within genomes of A.Br.008/009 canSNP clade strains of *B. anthracis*. By this approach, additional 32 strains could be assigned to five of ten defined clades.

## 1. Introduction

*Bacillus anthracis*, the causative agent of the zoonotic infectious disease anthrax has a broad geographic distribution, mostly affecting regions in Africa, South America and Asia [1]. Enzootic areas can, however, also be identified in Europe, North America [2] and Australia [3]. While rare in most of Europe, including Northern Europe [4,5] and Germany [6], the disease frequently reemerges in parts of Italy [7] and France [8]. The global population structure of *B. anthracis* is divided into three major canonical clades (branches), namely the A-, B- and C-branches [9]. Among these, the A-branch (A.Br.) is the most wide-spread, with the majority of isolates belonging to this lineage of the pathogen. Within the A-branch, the A.Br. 008/009 clade, i.e., strains phylogenetically defined by the two canonical (can) single nucleotide polymorphisms (SNPs) A.Br.008 and A.Br.009, is the one with the broadest geographical distribution. This clade has thus also been named the Trans-Eurasian branch [10] because it is dominant in most of Europe and (western) regions of Asia, even though we know today that some African [11] or American strains [8] are also included. It is currently unclear if, or when, such strains have been secondarily transported from Eurasia to other continents by human activities.

Recently, the phylogeny of *B. anthracis* in general, and also that of canSNP clade A.Br.008/009, has seen a major revision [12], expanding on the original genomic SNP-based typing scheme [9]. More than 10 minor yet distinct sub-clades have been defined, some of which have been nicknamed by an eponymous isolate or a common origin (such as A.Br.Pasteur or A.Br. Heroin), in addition to a systematic nomenclature defined by SNP-positions (A.Br.127 or A.Br.161) [12]. About half of the described strains in this seminal work [12] were grouped within the A.Br.008/009 clade, clearly emphasizing the clade’s worldwide significance.

With the objective of generating an increasingly detailed picture of the phylogenetic population structure of the Trans-Eurasian A.Br.008/009 canSNP clade of *B. anthracis*, we have now analyzed 35 strains from our strain collections on a genomic level. This new information was used to describe the phylogenetic placement of these isolates within the A.Br.008/009 clade, and to design and test new SNP-based assays. 

## 2. Materials and Methods 

### 2.1. Growth of B. anthracis and Extraction of DNA from Inactivated Culture Material

All live *B*. *anthracis* strains were handled in biosafety level 3 (BSL-3) laboratories at the Bundeswehr Institute of Microbiology (IMB) or University of Hohenheim. Cultures of *B. anthracis* from the strain collections (Table S1) were grown on blood agar and then chemically inactivated before further use [13]. DNA was isolated using MasterPure™ Gram Positive DNA Purification kit (Lucigen Middleton, WI 53562, USA) or DNeasy Blood and Tissue kit (Qiagen, Hilden, Germany), as described for Gram-positive bacteria. DNA concentrations were quantified using the Qubit dsDNA HS Assay Kit (Thermo Fisher Scientific, Dreieich, Germany) according to the supplier’s protocol. DNA preparations were stored at −20 °C until further use.

### 2.2. Whole Genome Sequencing

Whole genome sequencing was performed using the Illumina MiSeq platform with 2 × 300 bp v3-chemistry. For this, a compatible library using NEBNext® Ultra™ II DNA Library Prep Kit (New England Biolabs, Frankfurt am Main, Germany) with 100 ng of input-DNA was prepared. High-quality paired-end reads (Q > = 30) were assembled de novo using an in-house script based on the SPAdes (version 3.11.1) assembler [14] to create draft genomes, and Pilon (version 1.22) [15] for correcting SNPs or closing small gaps and INDELs. The obtained scaffolds were manually checked for contaminant reads and annotated automatically by the NCBI Prokaryotic Genome Annotation Pipeline [16] after submission. All data generated or analyzed during this study are included in this published article, and its supplementary information files are publically available in the NCBI Sequence Read Archive (SRA) repository (Bioproject PRJNA309927).

### 2.3. Analysis of Whole Genome Sequencing Data–SNP Calling

For rapid core chromosome multiple-alignment, the Parsnp tool from the Harvest Suite was used [17]. For this, a chromosome-dataset, representing genomes from public databases (Table S1) and the newly sequenced strains of *B. anthracis*, were aligned against the chromosome of *B. anthracis ‘Ames ancestor’* (NC_007530) as a phylogenetic outgroup using Parsnp (parameters -c -e -u -C 1000). To export the identified SNP-positions, HarvestTools (version 1.0) from the same software suite was used to create a vcf-(Variant Calling File) listing all SNP-positions. In order to enhance data quality, chromosome regions with closely adjacent SNPs (<10 bp distance), as well as positions harboring undefined nucleotides (“N”), were removed. This curated vcf-file was used as an input for HarvestTools to compile a multi-FASTA file out of the chromosome-dataset, comprising the concatenated SNPs as a multiple-sequence alignment. This concatenated sequence information was used to calculate a Maximum Likelihood tree in MEGA 7 [18,19]. The SNPs identified within the analyzed *B. anthracis* chromosomes are compiled in Table S2. In addition, a minimum spanning tree was computed in BioNumerics 6.6 (Applied Maths, Sint-Martens-Latem, Belgium) from the vcf SNP-file (in binary format) as input, and manually edited (using Powerpoint, Microsoft) for style.

### 2.4. Interrogation of SNPs via PCR by Relative Ct-Value Analysis (Delayed Mismatch Amplification Assay, DMAA)

In order to validate clade-specific SNPs identified by whole genome SNP-discovery, and to determine the distribution of these SNPs in additional *B. anthracis* DNAs, novel PCR assays were designed and tested. The new assay method is a probe-less real-time qPCR hybrid based on the Melt-MAMA test [20], previously used by us [4,13] or others [21], and an older melt-independent approach employing two separate PCR-reactions to interrogate for alternate allele states, ARMS (Amplification Refractory Mutation System) [22]. Our hybrid method makes use of mismatch (forward) primers featuring an additional mismatch to both alleles at the antepenultimate (-3) position [20] and the allele-specific match/mismatch at the 3’-position like in melt-MAMA and ARMS. These primers were then individually paired with a common reverse primer for real-time amplification with intercalating dye SYBR green of amplicons of approximately 100 bp in two separate reactions. Cycle threshold values (*C*t-values) were recorded and compared between paired reactions. The respective lower *C*t-value indicated preferred amplification; thus, the allelic state of the forward primer determined the scored SNP state (ancestral, ANC or derived, DER). This was quantified by subtracting the Ct-value of the PCR ANC forward primer from that of the PCR with DER forward primer. Thus, positive numeric values indicate ANC, and negative values DER allelic states. DMAA SNP-primer oligonucleotides for new SNPs and established canSNPs were designed surrounding the SNP positions with the Primer-BLAST tool of NCBI [23] using the *B. anthracis* Ames ‘Ancestor’ chromosome (accession # NC_007530) as a reference. DMAA-SNP primer sequences for real-time PCR-assays are listed in Table S3. Each primer pair was used in a 20 μL single-plex reaction. For this, 1 μM of each primer pair, and approximately 20 ng of the template DNA were added to 1 × LightCycler 480 High Resolution Melting Master mix (Roche, Mannheim, Germany). Amplification and analysis of Ct-values was carried out on a LightCycler 480 II instrument (Roche, Mannheim, Germany) as described in [13], without melting curve analysis.

### 2.5. Multi Locus VNTR (Variable Number of Tandem Repeats) Analysis (MLVA)

MLVA was done as described previously [24]. Briefly, amplification of the fragments of 31 marker-loci was performed in seven multiplex-PCRs. The fragment mixtures were analyzed on a Genetic Analyzer (ABI 3130, Applied Biosystems, Darmstadt, Germany) using either MegaBACE TMET (GE Healthcare, Solingen, Germany), Genescan 1200 LIZ (Applied Biosystems Darmstadt, Germany) or MapMarkerH 1000 (BioVentures, Murfreesboro, TN, USA) as size standards. The data were analyzed with GeneMapper TM software (Applied Biosystems, Darmstadt, Germany). The raw data of fragment lengths were normalized by codes, reflecting the actual copy numbers of the repeat sequences where possible. MLVA data analysis was performed using cluster analysis of categorical coefficients. Herein, a similarity tree based on the unweighted pair group method with arithmetic mean (UPGMA) method was computed in BioNumerics 6.6 (Applied Maths, Sint-Martens-Latem, Belgium) and manually edited (using Powerpoint, Microsoft) for style.

## 3. Results and Discussion

### 3.1. Assignment of Newly Sequenced Strains to canSNP Groups of B. anthracis

**Figure 1:**
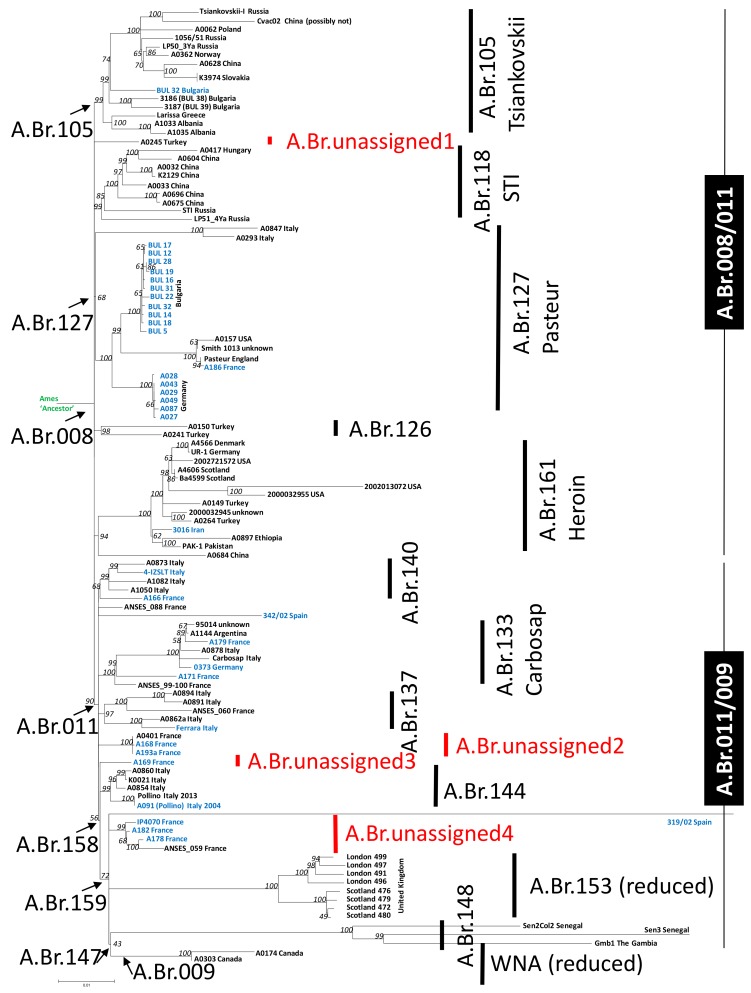
Rooted phylogenetic tree of representatives of the A.Br.008/009 canonical single-nucleotide polymorphisms (canSNP) clade of *B. anthracis* derived from chromosomal SNPs. A total of 3754 chromosomal SNPs were used to construct a Maximum Likelihood tree (bootstrap confidence values from 500 permutations were generated and the tree with the highest likelihood is shown). The isolate names and countries of origin are indicated at branch termini (blue: sequenced in this study; black: sequences from databases; red branch labels: new unassigned sub-branches). Vertical bars and arrows designate canSNP clades and sub-clades. The tree is rooted to the *B. anthracis* reference strain Ames ‘Ancestor’, which belongs to the A.Br.Ames clade.

From the mapping of 35 newly sequenced *B. anthracis* strains and 75 chromosomal sequences of the A.Br.008/009 clade isolates from public databases against the Ames ‘Ancestor’ reference genome, a total of 3754 chromosomal SNP positions were identified. Figure 1 illustrates a Maximum Likelihood tree deduced from this SNP data. 

The Ames ‘Ancestor’ genome served as the root of this tree and is at a 200 SNP distance from the basal node of the A.Br.008/009 polytomy (Figure 1). The assignment of sequenced chromosomes within the established A.Br. sub-clades agrees with previous work [12], further populating most of the minor sub-branches. The new chromosomes were placed within the global A.Br.008/009 phylogeny as follows. Bulgarian strains, originally described based on MLVA and canSNP data in [10], were distributed in clades A.Br.105 (Tsiankovskii) and A.Br.127 (Pasteur). New French strains belonged to lineages A.Br.127 (Pasteur), 140, 133 (Carbosap) or to new minor sub-group branches originating from the A.Br.158 or A.Br.159 polytomies. Both of these minor branches had previously included a single French isolate [12]. A single Iranian isolate (3016) expands the diversity of the A.Br.161 (Heroin) clade and a single Italian isolate Ferrara belonged to A.Br.140. German isolates were attributed to A.Br.127 (Pasteur) and A.Br.133 (Carbosap). The two Spanish isolates [25] were rather unique. Strain 319/02 branched off the A.Br.159 polytomy, whereas strain 342/02 branched off the A.Br.011 polytomy. No new isolates were added through this work to clades A.Br.118 (STI), 126, 137, 153 and 009 (WNA) (Figure 1).

### 3.2. Relationships of Newly Sequenced Strains Within canSNP Groups Sub-Lineages of B. anthracis

**Figure 2:**
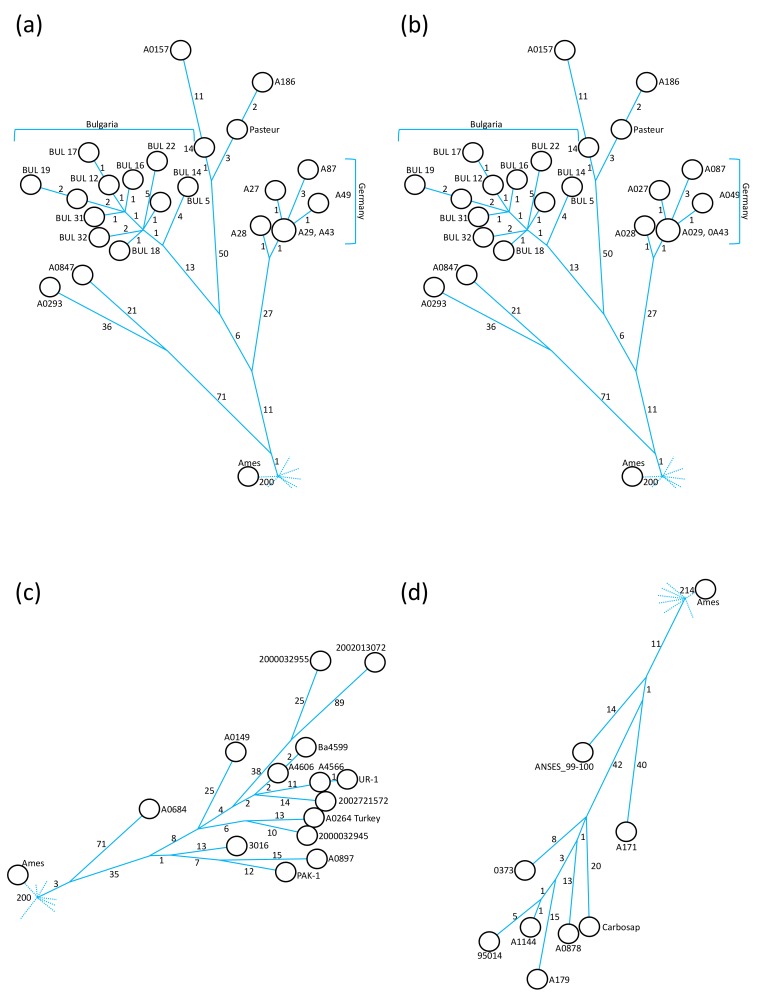
Minimum-spanning trees of representative A.Br.008/009 sub-clades of the canSNP clade of *B. anthracis* derived from chromosomal SNPs. Shown are numerical SNP differences between genomes of *B. anthracis* isolates (from Figure 1) belonging to four different prominent A.Br.008/009 sub-clades (**a**) A.Br.105 (Tsiankovskii), (**b**) A.Br.127 (Pasteur), (**c**) A.Br.161 (Heroin) and (**d**) A.Br.133 (Carbosap). Indicated by dotted lines are the positions of polytomies at the bases of these sub-clades. **a**–**c** indicate the SNP distance from the A.Br.008 or (**d**) the A.Br.011 polytomy to the reference chromosome Ames ‘Ancestor’.

Further analysis of the A.Br.008/009 canSNP clade by individual sub-branches comprising newly sequenced chromosomes allows for additional observations. Only one of the newly sequenced strains, BUL 40 from Bulgaria, grouped within the A.Br.105 (Tsiankovskii) clade. This isolate was located at a distance of 41 autapomorphous (i.e., isolate-specific, derived characters) SNPs from the polytomy leading to A.Br.105 (Figure 2A) and formed the sole isolate of a sister branch to that leading to the eponymous Tsiankovskii strain. 

Notably, the closest relative of strain BUL 40 (63 SNPs distance) was strain LP50_3Ya, isolated from Yakutia in Russia (Figure 2a), rather than the two other Bulgarian strains (BUL 19 and BUL 39) from our collections that had been genome sequenced previously [12]. An additional genome 81/1 from Russia [26], which was not part of our analysis in Figure 1, also branched of the internal A.Br.105 polytomy. Overall, the A.Br.105 clade exhibits a strong dominance of isolates from eastern European countries (Figure 2a).

Within clade A.Br.127 (Pasteur), the German isolates formed a closely related group (Figure 1), exhibiting a maximum of five SNP differences within this group (Figure 2b). Strains A029 (1954) and A043 (1950ies) were SNP-identical. Both strains belonged to a historical collection of the Veterinary Faculty of Giessen University. However, as their MLVA types are different (see below) these strains may have been isolated from different outbreaks. The German A.Br.127 group was separated by 39 synapomorphous SNPs from the A.Br.008 polytomy, with one SNP (pos. 72,398) defining the entire A.Br.127 clade (Figure 2b and Table S2). The Bulgarian sub-clade was 19 SNPs apart from the node leading to the group of German isolates. Within the 11 Bulgarian chromosomes, there was a maximum of 10 SNPs separating these closely related chromosomes (Figure 2b). Of the 13 synapomorphous SNPs specific for the Bulgarian clade of A.Br.127, four were unique (pos. 189,687; 611,969; 4,437,026 and 4,525,831) and nine have been defined previously as A.Br.130 [12] (pos. 99,992; 1,235,601; 1,287,115; 3,564,952; 3,725,610; 3,930,606; 4,595,281; 4,607,259 and 5,109,459). The additional five SNPs belong to the A.Br.130 only (pos. 2,139,594; 2,540,931; 2,559,376; 4,974,063 and 5,050,288) (Table S2). Overall, this A.Br.127 (Pasteur) clade has been considerably populated by newly sequenced strains. The group of closely related Bulgarian strains isolated in the mid-twentieth century formed a rather deep-branching sub-clade within A.Br.127. An even deeper-branching sub-clade from roughly the same time period was exclusively made of German isolates (Figure 2b).

Strain A166 (France) of clade A.Br.140 (Figure 1) differed from strain 4-IZSLT (Italy) by 57 apomorphous SNPs (Table S2). The closest relative of strain A166 was strain A1050 (47 SNPs distance, Italy) and the closest relative of strain 4-IZSLT was strain A0873 (25 SNPs distance, Italy). The largest distance within the A.Br.140 canSNP clade was between strains A1082 and A166 (59 SNPs; Table S2). Strain A166 was SNP-identical to the previously published strain ANSES_089 and showed only one SNP difference to strain ANSES_087 [8] (Table S2), suggesting these strains originated from the same or closely related outbreak events.

Clade A.Br. 161 (Heroin) was separated from the A.Br.008 polytomy by three synapomorphous SNPs (pos. 1,600,499; 1,979,090 and 5,013,862; Table S2; Figure 2c). Strain 3016, from Iran, is not particularly closely related to the strains associated with injectional anthrax outbreaks in Europe from 2000 until 2013 [27], as the strain 3016 chromosome was separated by 30 to 41 SNPs from outbreak strains (Figure 2c). Instead, strain 3016 shared a common hypothetical ancestor with strains A0897 (Ethiopia; 36 SNPs distance) and PAK-1 (Pakistan; 32 SNPs distance), respectively (Figure 1 and Figure 2c).

Three new strains were added to the A.Br.133 (Carbosap) clade (Figure 1 and Figure 2d). Strain 0373 (Germany) was part of an internal polytomy within A.Br.133. This chromosome featured the shortest branch length (eight apomorphous SNPs from node) of this sub-clade and that of strain A179 (19 SNPs from node, France) had the longest (Figure 2d). Another chromosome of this internal polytomy was that of the eponymous Carbosap strain from Italy (20 SNPs from node). An additional French isolate (A171) was positioned 52 SNPs away from the polytomy leading to A.Br.133 (Figure 2d) and branched off earlier than strains A179 and 0373 (these three strains shared 12 SNPs within the A.Br.133 clade).

An unassigned lineage (A.Br.unassigned2 in Figure 1) with single isolate A0401 as the sole previous member is now accompanied by a SNP-identical isolate (A193a) and one isolate differing by one SNP (A168) (Table S2; Figure 1). While strain A0401 (alternate IDs CNEVA 7193 or K1895) has incomplete metadata, except that it was isolated from a cow in France [12], SNP-identical strain A193a is also from France and was isolated in 1997, from cattle. Possibly, strains A0401 and A193a represent a single isolate or, alternatively, were sampled from a common outbreak event (compare the “twin Pollino” isolates below). A third strain (A168) is from France as well and was isolated from the Haute Marne region in 1998. This strain shared 23 of 24 SNPs with strains A0401/A193a from the node that separated this minor clade from the A.Br.011 polytomy (Table S2). Previously published strain ANSES_122 was SNP-identical to strain A193a and showed only one SNP difference to strain A168 [8] (data not shown), again suggesting these strains originated from the same or closely related outbreak events.

A.Br.unassigned3 features a single strain A169 from France, which was wedged between canSNP A.Br.158 and A.Br.159 (Figure 1). The isolate was part of the A.Br.158 polytomy, from which it was separated by 21 apomorphous SNPs. This isolate was distinct from a group of Italian strains by canSNP A.Br.144. Of this clade, strain A0854 was the closest relative of strain A169 (38 SNPs distance; Table S2).

Strain IP4070 was the most basal isolate (18 SNPs from node; Table S2) in the A.Br.unassigned4 clade (Figure 1), which branched off from the A.Br.159 polytomy (11 synapomorphous SNPs distance) and has comprised only French isolates to date. Strain IP4070 was separated from strain A182 by 10 (the smallest distance within this clade), from A178 by 19 and from ANSES_059 by 33 SNPs (the largest distance within this clade). Strain A182 differed from strain A178 by 13 SNPs and from ANSES_059 by 27 SNPs. It is likely that strain IP4070 and previously published strain ANSES_094 [8] originated from the same or closely related outbreak events, as both were SNP-identical (data not shown). The same possibility applies to strains A182 and ANSES_032 (data not shown). The closest relative to this clade from a neighboring clade was strain A0854 (35 SNPs, canSNP group A.Br.144; Table S2).

Clade A.Br.144 of purely Italian samples (Figure 1) is included in our analysis because we sequenced strain A091 from Pollino national park in Italy. This isolate originated from an outbreak of anthrax among cattle on a mountain meadow in 2004 (described in [13]). At that time, isolate A091 was taken and stored. An international team revisited the location in 2014 and retrieved additional samples from the on-site buried remains of the animal [13]. Notably, both isolates are SNP identical, notwithstanding the 10 year gap of sampling, though we have observed some genetic diversity when analyzing multiple sampling from the same buried carcass [13]. Thus, this is a rare example of genomically comparing temporally separated samples from the same diseased host.

The properties of our two Spanish strains within the A.Br.008/009 phylogeny are quite peculiar. Both strains exhibited long unique branches, that either reflect an extended evolutionary history or nucleotide-changes caused by an increased mutation rate (Figure 1). Both strains, 319/02 and 342/02, grouped within clade A.Br.011, but at different positions. Strain 319/02 separated 379 apomorphous SNPs from the A.Br.159 polytomy. Possibly, this isolate is a mutator strain, as described before for another strain with a similar enormous number of phylogenetically uninformative SNP-positions [27]. On the other hand, strain 319/02 was 373 SNPs and strain Sen3 359 SNPs, apart from their hypothetical last common ancestor (Table S2). So, alternatively, these large numbers of autapomorphous characters may indicate real evolutionary changes in this clade. It will therefore be interesting to compare these isolates with yet-uncharacterized isolates from the Iberian Peninsula and West Africa. 

After the split from the A.Br.159 polytomy, Spanish strain 319/02 and the clade leading to A.Br. WNA shared two basal SNPs: The A.Br.147 canSNPs (SNP pos. 1,776,654 and SNP 2,917,555; Table S2). Strain 319/02 also shared four synapomorphous SNPs (Table S2), with strains Gmb1 (The Gambia), Sen2Col2 and Sen3 (Senegal). Notably, this is not reflected in the phylogenetic representation in Figure 1. There, these lineages are depicted as separated (ancestral) by A.Br.147. This A.Br.147 branch, however, is only supported by 43%. The derived allelic state for SNP A.Br.147 (pos. 1,776,654) was also independently confirmed (see below DMAA PCR). Thus, mindful of the caveats described above, strain 319/02 was the closest European isolate to these three African strains in this study (632 SNPs between strains 319/02 and Sen2Col2; Table S2). Conversely to strain 319/02, the other Spanish strain 342/02 branched off directly from the A.Br.011 polytomy, from which the 342/02 chromosome was separated by 107 SNPs (Figure 1). The closest relatives of strain 342/02 were strains ANSES_088 (123 SNPs), A169 (129 SNPs) and A193a/A0401 (130 SNPs) (Table S2).

### 3.3. Relationships of Newly Sequenced B. anthracis Strains Drawn From MLVA Typing

**Figure 3:**
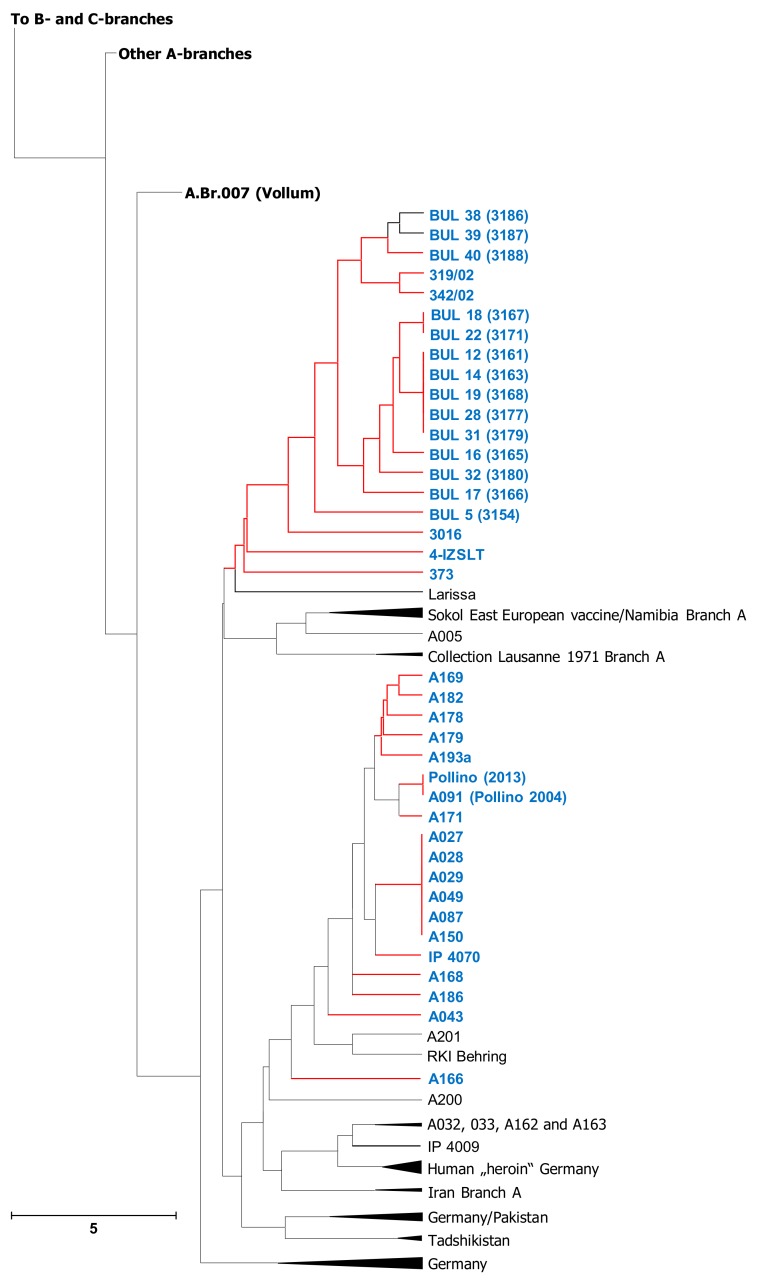
Unweighted pair group method with arithmetic mean (UPGMA) cluster analysis based on multilocus variable number of tandem repeat analysis (MLVA)-data for *B. anthracis* A.Br.008/009 clade strains and their relatives. The relationships of 1002 *B. anthracis* isolates, based on analysis using the MLVA-31 typing scheme, are shown. Clustering of the strains’ profiles was done using a categorical coefficient, thus, distances between circles do not necessarily reflect accurate phylogenetic distances. Strains genotyped in this study are presented individually (genome-sequenced strains in this study labeled in blue with red branches) alongside their closest relatives, whereas additional isolates are grouped and represented as triangles.

Though SNP-typing provides the highest phylogenetic resolution power to date, MLVA typing is an established methodology for the classification of *B. anthracis* isolates. We therefore offer an additional presentation of our SNP-based phylogenetic strain analysis by MLVA typing, in order to make possible backward-compatibility. For this, we grouped our newly sequenced strains with previously MLVA-typed isolates, so that this new data can be easily related with published MLVA-typing studies of the anthrax pathogen (Figure 3 and Table S4). 

In comparison to SNP-based analysis, MLVA cluster analysis presented a variety of interesting and possibly important differences. In MLVA (Figure 3), strain A043 was markedly different to strains A027, A028, A029, A049 and A087. Conversely, in the SNP-based tree (Figure 1), these genomes were very closely related. Strains A168 and A193a were SNP-identical but clearly separated by MLVA. Similarly, strains BUL 32 and BUL 22 were almost SNP-identical but MLVA-different. The same applies to strains BUL 12 and BUL 17, as well as BUL 14, BUL 18, BUL 22 and BUL 32. Of the latter four, only BUL 18 and BUL 32 share a MLVA-type; the other two were clearly separated. Finally, the genetically very different Spanish isolates 319/02 and 342/02 (Figure 1) differed only by one MLVA-marker (Figure 3).

Thus, it appears that in some instances there is conflicting information between typing information gained by MLVA or SNP-analysis, respectively. This might be explained by the fact that MLVA is not a strict phylogenetic approach, but rather a tool to elucidate population structure by interrogating for categorical differences in individually evolved repeat numbers (which might result in size homoplasy) [28]. In other instances, MLVA-31 may achieve higher differentiation between closely related isolates than chromosome-wide SNP-analysis. Therefore, it would certainly make sense to continue using MLVA data for genomic strain-typing. Currently, an in silico approach from sequence reads is still error-prone, due to the intrinsically repetitive nature of MLVA markers and the possible failure to accurately assemble such regions [29], especially the long ones present in *B. anthracis* (unpublished observation). In the near future, however, with the increasing use of long-read sequencing technologies (such as nanopore sequencing), this hurdle certainly will disappear.

### 3.4. New SNP-PCR Discriminating Assays for Phylogenetic Placement of Additional A.Br.008/009 Clade Strains of B. anthracis

Newly identified SNP positions from our genome data analysis (FS2), which were considered potentially useful for grouping additional, non-genome-sequenced *B. anthracis* strains of the A.Br.008/009 canSNP lineage into relevant sub-clades from Figure 1, were developed into SNP-discriminating DMAA PCR assays (Table S2). These assays were then used to interrogate the SNP states of an additional 32 strains (Table 1). 

Of three A.Br.118 (STI) strains, all were, indeed, different instances of the STI vaccine strain in our collection. A single additional A.Br.Pasteur isolate was from France. All 13 identified A.Br.Pasteur-BUL strains were from Bulgaria [10]. However, not all Bulgarian strains belonged to this canSNP group, for nine strains grouped within canSNP A.Br147, and one within A.Br.105. This mirrors earlier results, since these Bulgarian strains have already been differentiated based on MLVA typing [10]. In this earlier work (what we now know as) canSNP A.Br.105 isolates were designated as genotype (GT) 1, 2 and 2a. 

Further, strains from our collection, typed by DMAA, were from Germany, Bulgaria or Namibia (Table 1). Given that Namibia has been a colony of the German Reich (from 1884 until 1919) it is possible that this Namibian isolate might have originated from infected livestock transport from Central Europe to Namibia.

Besides the ease of implementation, there is an additional advantage of the DMAA PCR SNP-typing approach to methods based on melt-temperature analysis. This is, DMAA can differentiate DER/ANC pyrimidine or purine pairs (such as T/A in DMAA canSNP 161; Table S3) where differentiation is not readily possible by Melt-MAMA [20] or HRM-typing [30].

In conclusion we analyzed and refined the phylogenetic population structure of the *B. anthracis* A.Br.008/009 (or Trans-Eurasian) canSNP lineage which has worldwide significance. From 35 newly and 75 published genomes we designed and tested new SNP-based PCR-assays which allowed attributing additional 32 strains to various sub-clades within the A.Br.008/009 canSNP lineage.

## Figures and Tables

**Table 1:** SNP-typing of additional *B. anthracis* strains by DMAA PCR.

SNP Designation	# of Strains	Origin (Year of Isolation)
A.Br.105^1^ (Tsiankovskii)	4	Germany (unknown), Switzerland (unknown), Bulgaria (1960–1980), Namibia (unknown)
A.Br.118 (STI) ^1^	3	Russia (1991), 2 × Russia (unknown)
A.Br.127 (Pasteur) ^1^	1	France (1996)
A.Br.Pasteur-GER	0	/
A.Br.Pasteur-BUL	13	Bulgaria (1960–1980)
A.Br.133 (Carbosap) ^1^	0	/
A.Br.NN4	0	/
A.Br.140 ^1^	0	/
A.Br.147 ^1^	11	9 x Bulgaria (1960–1980), Germany (2007), Canada (unknown)

^1^ previously defined SNPs [12].

## Supplementary Materials

The following are available online at https://www.mdpi.com/2076-2607/7/12/689/s1.

## References

[1] WHO (2008). World Health Organization Anthrax in Humans and Animals.

[2] Blackburn J.K., McNyset K.M., Curtis A., Hugh-Jones M.E. (2007). Modeling the geographic distribution of *Bacillus anthracis* the causative agent of anthrax disease, for the contiguous United States using predictive ecological niche modeling. Am. J. Trop. Med. Hyg..

[3] Barro A.S., Fegan M., Moloney B., Porter K., Muller J., Warner S., Blackburn J.K. (2016). Redefining the Australian anthrax belt: Modeling the ecological niche and predicting the geographic distribution of *Bacillus anthracis*. PLoS Negl. Trop. Dis..

[4] Lienemann T., Beyer W., Pelkola K., Rossow H., Rehn A., Antwerpen M., Grass G. (2018). Genotyping and phylogenetic placement of *Bacillus anthracis* isolates from Finland, a country with rare anthrax cases. BMC Microbiol..

[5] Ågren J., Finn M., Bengtsson B., Segerman B. (2014). Microevolution during an anthrax outbreak leading to clonal heterogeneity and penicillin resistance. PLoS ONE.

[6] Antwerpen M., Elschner M., Gaede W., Schliephake A., Grass G., Tomaso H. (2016). Genome sequence of *Bacillus anthracis* strain Stendal, isolated from an anthrax outbreak in cattle in Germany. Genome Announc..

[7] Manzulli V., Fasanella A., Parisi A., Serrecchia L., Donatiello A., Rondinone V., Caruso M., Zange S., Tscherne A., Decaro N. (2019). Evaluation of in vitro antimicrobial susceptibility of *Bacillus anthracis* strains isolated during anthrax outbreaks in Italy from 1984 to 2017. J. Vet. Sci..

[8] Vergnaud G., Girault G., Thierry S., Pourcel C., Madani N., Blouin Y. (2016). Comparison of French and worldwide *Bacillus anthracis* strains favors a recent, post-Columbian origin of the predominant North-American clade. PLoS ONE.

[9] Van Ert M.N., Easterday W.R., Huynh L.Y., Okinaka R.T., Hugh-Jones M.E., Ravel J., Zanecki S.R., Pearson T., Simonson T.S., U’Ren J.M. (2007). Global genetic population structure of *Bacillus anthracis*. PLoS ONE.

[10] Antwerpen M., Ilin D., Georgieva E., Meyer H., Savov E., Frangoulidis D. (2011). MLVA and SNP analysis identified a unique genetic cluster in Bulgarian *Bacillus anthracis* strains. Eur. J. Clin. Microbiol. Infect. Dis..

[11] Pullan S.T., Pearson T.R., Latham J., Mason J., Atkinson B., Silman N.J., Marston C.K., Sahl J.W., Birdsell D., Hoffmaster A.R. (2015). Whole-genome sequencing investigation of animal-skin-drum-associated UK anthrax cases reveals evidence of mixed populations and relatedness to a US case. Microb. Genom..

[12] Sahl J.W., Pearson T., Okinaka R., Schupp J.M., Gillece J.D., Heaton H., Birdsell D., Hepp C., Fofanov V., Noseda R. (2016). *Bacillus anthracis* genome sequence from the Sverdlovsk 1979 autopsy specimens. mBio.

[13] Braun P., Grass G., Aceti A., Serrecchia L., Affuso A., Marino L., Grimaldi S., Pagano S., Hanczaruk M., Georgi E. (2015). Fasanella A Microevolution of anthrax from a young ancestor (MAYA) suggests a soil-borne life cycle of *Bacillus anthracis*. PLoS ONE.

[14] Bankevich A., Nurk S., Antipov D., Gurevich A.A., Dvorkin M., Kulikov A.S., Lesin V.M., Nikolenko S.I., Pham S., Prjibelski A.D. (2012). Pevzner PA SPAdes: A new genome assembly algorithm and its applications to single-cell sequencing. J. Comput. Biol..

[15] Walker B.J., Abeel T., Shea T., Priest M., Abouelliel A., Sakthikumar S., Cuomo C.A., Zeng Q., Wortman  J., Young S.K. (2014). Pilon: An integrated tool for comprehensive microbial variant detection and genome assembly improvement. PLoS ONE.

[16] Angiuoli S.V., Gussman A., Klimke W., Cochrane G., Field D., Garrity G., Kodira C.D., Kyrpides N., Madupu R., Markowitz V. (2008). Toward an online repository of Standard Operating Procedures (SOPs) for (meta)genomic annotation. OMICS.

[17] Treangen T.J., Ondov B.D., Koren S., Phillippy A.M. (2014). The Harvest suite for rapid core-genome alignment and visualization of thousands of intraspecific microbial genomes. Genome Biol..

[18] Tamura K., Nei M. (1993). Estimation of the number of nucleotide substitutions in the control region of mitochondrial DNA in humans and chimpanzees. Mol. Biol. Evol..

[19] Kumar S., Stecher G., Tamura K. (2016). MEGA7: Molecular Evolutionary Genetics Analysis version 7.0 for bigger datasets. Mol. Biol. Evol..

[20] Birdsell D.N., Pearson T., Price E.P., Hornstra H.M., Nera R.D., Stone N., Gruendike J., Kaufman E.L., Pettus A.H., Hurbon A.N. (2012). Melt analysis of mismatch amplification mutation assays (Melt-MAMA): A functional study of a cost-effective SNP genotyping assay in bacterial models. PLoS ONE.

[21] Kreizinger Z., Sulyok K.M., Makrai L., Ronai Z., Fodor L., Janosi S., Gyuranecz M. (2016). Antimicrobial susceptibility of *Bacillus anthracis* strains from Hungary. Acta Vet. Hung..

[22] Newton C.R., Graham A., Heptinstall L.E., Powell S.J., Summers C., Kalsheker N., Smith J.C., Markham A.F. (1989). Analysis of any point mutation in DNA. The amplification refractory mutation system (ARMS). Nucleic Acids Res..

[23] Ye J., Coulouris G., Zaretskaya I., Cutcutache I., Rozen S., Madden T.L. (2012). Primer-BLAST: A tool to design target-specific primers for polymerase chain reaction. BMC Bioinform..

[24] Beyer W., Bellan S., Eberle G., Ganz H.H., Getz W.M., Haumacher R., Hilss K.A., Kilian W., Lazak J., Turner W.C. (2012). Distribution and molecular evolution of *Bacillus anthracis* genotypes in Namibia. PLoS Negl. Trop. Dis..

[25] Bassy O., Jimenez-Mateo O., Ortega M.V., Granja C., Cabria J.C. (2018). Rapid identification of *Bacillus anthracis* by real-time PCR with dual hybridization probes in environmental swabs. Mol. Cell. Probes.

[26] Pisarenko S.V., Eremenko E.I., Ryazanova A.G., Kovalev D.A., Buravtseva N.P., Aksenova L.Y., Evchenko A.Y., Semenova O.V., Bobrisheva O.V., Kuznetsova I.V. (2019). Genotyping and phylogenetic location of one clinical isolate of *Bacillus anthracis* isolated from a human in Russia. BMC Microbiol..

[27] Keim P., Grunow R., Vipond R., Grass G., Hoffmaster A., Birdsell D.N., Klee S.R., Pullan S., Antwerpen M., Bayer B.N. (2015). Whole genome analysis of injectional anthrax identifies two disease clusters spanning more than 13 years. EBioMedicine.

[28] Estoup A., Jarne P., Cornuet J.M. (2002). Homoplasy and mutation model at microsatellite loci and their consequences for population genetics analysis. Mol. Ecol..

[29] Nadon C., Van Walle I., Gerner-Smidt P., Campos J., Chinen I., Concepcion-Acevedo J., Gilpin B., Smith A.M., Man Kam K., Perez E. (2017). PulseNet International: Vision for the implementation of whole genome sequencing (WGS) for global food-borne disease surveillance. EuroSurveillance.

[30] Fortini D., Ciammaruconi A., De Santis R., Fasanella A., Battisti A., D’Amelio R., Lista F., Cassone A. (2007). Carattoli A Optimization of high-resolution melting analysis for low-cost and rapid screening of allelic variants of *Bacillus anthracis* by multiple-locus variable-number tandem repeat analysis. Clin. Chem..

